# Extending MAM5 Meta-Model and *JaCalIV E* Framework to Integrate Smart Devices from Real Environments

**DOI:** 10.1371/journal.pone.0149665

**Published:** 2016-02-29

**Authors:** J. A. Rincon, Jose-Luis Poza-Lujan, V. Julian, Juan-Luis Posadas-Yagüe, C. Carrascosa

**Affiliations:** 1 Universitat Politècnica de València, Departamento de Sistemas Informáticos y Computación (DSIC), Camino de Vera s/n, Valencia, Spain; 2 Universitat Politècnica de València, Institute of Control Systems and Industrial Computing (ai2), Camino de Vera s/n, Valencia, Spain; Nankai University, CHINA

## Abstract

This paper presents the extension of a meta-model (*MAM*5) and a framework based on the model (*JaCalIVE*) for developing intelligent virtual environments. The goal of this extension is to develop augmented mirror worlds that represent a real and virtual world coupled, so that the virtual world not only reflects the real one, but also complements it. A new component called a smart resource artifact, that enables modelling and developing devices to access the real physical world, and a human in the loop agent to place a human in the system have been included in the meta-model and framework. The proposed extension of *MAM*5 has been tested by simulating a light control system where agents can access both virtual and real sensor/actuators through the smart resources developed. The results show that the use of real environment interactive elements (smart resource artifacts) in agent-based simulations allows to minimize the error between simulated and real system.

## Introduction

The emergence of new virtual technologies such as *HoloLens* (https://www.microsoft.com/microsoft-hololens/en-us), in which the world is augmented using virtual objects is a first step in the creation of an *augmented world*. *Augmented worlds* were defined by Gelernter [1] as “software models of some chunk of reality, some piece of the real world going on outside your windows.” Gelernter indicates that four keys are necessary to develop mirror worlds: a deep picture; a live picture; an agent; and history.

The *mirror world* is based on the concept of an *augmented world*, but in a *mirror world*, elements that enable high scalability are introduced. This *mirror world* integrates elements such as artificial intelligence (AI), augmented reality (AR), multi-agent systems (MAS) and mobile augmented reality. The design of this mirror world enables the creation of applications of ambient intelligence *AmI*, where the human may interact with virtual entities and these virtual entities may interact with the real world through sensors, robots, or any device that can connect with a human.

Software solutions that enforce autonomy, robustness, flexibility, and adaptability of a system under development are currently necessary. This is because the real world may always be changing and so software solutions need to change in real time. For this reason, these applications need to be robust enough to support a change in the environment and, at the same time, flexible enough to dynamically add elements.

Agent organizations that dynamically auto-adjust themselves to obtain advantages from their environment seem to be a suitable technology to cope with the development of this type of system. These organizations could appear in distributed and dynamic systems, such as grid domains, peer-to-peer networks, or other contexts where software agents dynamically group together to offer compound services as in intelligent virtual environments (IVE). An IVE is a virtual environment simulating a physical (or real) world inhabited by autonomous intelligent entities [2].

IVEs are addressed by a huge number of simultaneous entities, so they must be supported by highly scalable software. This software must also be able to adapt to changes in the number of entities and user needs. Current technology used to develop this kind of product lacks elements to facilitate the adaptation and management of the system. Traditionally, this type of application uses the client/server paradigm, but due to its features, a distributed approach such as multi-agent systems (MAS) seems compatible with the development of components that will evolve autonomously and coordinate with environmental evolution.

One approach for the development of these systems is the JaCalIVE framework. The JaCalIVE (http://jacalive.gti-ia.dsic.upv.es/)(Jason Cartago implemented Intelligent Virtual Environment) framework provides an agent-oriented method to develop IVEs along with a supporting platform to execute them. JaCalIVE is based on the MAM5 meta-model, which describes a method to design IVEs [3]. MAM5 is based in the A&A (agent &artifact) meta-model [4] that describes environments for MAS populated by agents and other entities that are called *artifacts*.

An IVE is composed of three important parts: artifacts, agents, and a physical simulation. Artifacts are the elements on which the environment is modelled. Agents are the IVE intelligent part. The physical simulation is in charge of giving the IVE the look of the real or physical world, enabling the simulation of physical phenomena such as gravity or collision detection.

This paper presents the extension of both the MAM5 meta-model and the JaCalIVE framework to model, design, and implement augmented worlds that more precisely mirror worlds. The concept of a smart resource artifact has been included in the meta-model in order to enable the design of the physical world in the environmental model. Moreover, the extended meta-model also enables the modelization of humans who can interact with other entities in the IVE. Finally, some experiments have been developed to validate the proposed extension. The example proposes the design of the optimal lighting for a road. To do this, the system tries to minimize the time that streetlights are on and the number of sensors needed to track driver itineraries.

Next, the most relevant methods, techniques, and technology used to devise mirror worlds are summarized. The starting point is a meta-model and framework for devising intelligent virtual environments. Then, it is commented on the *MAM*5 meta-model and *JaCalIVE* framework that enables the development of IVEs in MAS terms. Some comments about accessing the physical real environment and *smart resources* are then introduced.

### IVE

Currently, there is an increasing interest in the application of IVEs in a wide variety of domains. IVEs have been used to create advanced simulated environments [5–7] in domains such as: education [8], entertainment [9–12], e-commerce [13], health [14, 15] and VR-based simulations [16].

One of the key features of any IVE is that a high level of user immersion is offered. To achieve this it is necessary that the IVE has the ability to simulate physical conditions of the real world such as gravity, friction, and collisions. To increase graphical realism, physical simulators should include dynamic and static objects that populate the IVE in a three-dimensional environment. Relevant physical simulation tools include *JBullet*(http://jbullet.advel.cz/) and *Open Dynamic Engine (ODE)*(http://www.ode.org/).

Another important feature of any IVE is a high level of graphic realism. Currently, there are available some well-developed graphical simulators such as *Unity 3D*(http://unity3d.com/unity), *Unrealengine UDK*(http://www.unrealengine.com/udk/) y *Cryengine*(http://www.crytek.com/cryengine). Although these simulators were initially designed for videogames, they can be used to simulate IVEs.

In some IVEs, it is very important to get information of the real world. With this information, it is possible to know what is happening in the real world, allowing to create a complex simulation mixing the information of the real world and information emulated in the virtual world. This is also the idea behind augmented worlds or even mirror worlds.

In some IVEs, it is very important to obtain information from the real world. With this information, it is possible to create a complex simulation mixing information from the real world and information emulated in a virtual world. This is also the idea behind augmented and mirror worlds. This type of application must also take into account that a human is immersed in the loop—producing a double immersion [17]. This double immersion enables humans to interact and communicate with agents using natural human interfaces, while at the same time, agents perceive and communicate with humans and other agents. This kind of interaction enables the creation of a “*human agent society*”, a type of application where agents offer services to humans or to other agents in an integrated environment.

### Multi-Agent systems

Previously, it has been highlighted the importance of giving *realism* to IVEs so that users have the desired level of immersion. This realism is provided by physical simulation and 3D visualization, but this is only part of a virtual environment. To be an IVE, a virtual environment needs to give entities the intelligence to enhance the user’s immersion MAS is one of the most popular artificial intelligence technique for modeling IVEs.

This is mainly due to the characteristics that agents have, such as autonomy, proactivity, reactivity, and sociability. But this does not mean that no other AI techniques can be used within MAS for IVE development. An agent can include as a decision-making mechanism other algorithms that improve the deliberative process—such as: reinforcement learning [18]; genetic algorithms [19]; Markov models [20]; classification [21, 22]; and neuronal networks [23] or use any hybrid artificial intelligence system method [24].

However, when modeling an environment it is necessary to take into account that not all the entities are agents. The A&A meta-model [25, 26] describes a methodology for modeling environments using artifacts. Artifacts represent the first level of abstraction when modeling environments. This is mainly due to the clear differentiation among entities in systems of this kind. This differentiation can determine which items are objects (artifacts) and which are intelligent entities (agents).

The BDI model (belief—desire—intention) [27–29] is the best known and most frequently used agent model for designing intelligent agents. This model is based on logic and psychology, and creates symbolic representations of agent beliefs, desires, and intentions. The beliefs are the information the agent has about the environment. This information can be updated at each time step or not. The obsolescence of the used information forces the agent to perform deliberative processes. Desires are the actions that the agent could make. This does not mean that every desire of an agent must be performed. Finally, intentions represent the actions that the agent has decided to perform. These actions may be goals that have been delegated to the agent or may be the result of previous deliberation processes.

Different approaches have been devised to develop MAS. One of the first tools used for implementing agents is the JADE platform. JADE was used for the development of *JGOMAS (Game Oriented Multi -Agent System based on Jade)* [30–32]. JADE does not directly provide a BDI model but offers an extension called JADEX that enables developers to design BDI-oriented MAS that incorporate the representation of beliefs, desires and intentions. JADEX has been used for modeling environments such as that presented in [33]. *Jason* is another development tool used for MAS programming and also integrates the BDI model.

In this proposal Jason is employed as the programming toolkit for BDI agents [34]. The main reason for using JASON is its complete integration with CArtAgO (Common ARTifact infrastructure for AGents Open environments) [35]. CArtAgO is a framework/infrastructure for modeling artifacts that can run virtual environments. This framework allows the implementation of open work-spaces, which facilitate the creation of distributed environments.

### MAM5

MAM5 [3] is a model for designing IVEs that is based in the A&A meta-model. It is for use by IVE designers who want to design an IVE based on a multi-agent system. As it is intended to be distributed, the human interface part of the system is decoupled from the intelligent part (designed using MAM5). The fact that both parts are distributed facilitates development and gives more flexibility to the final applications (enabling different interfaces to be connected at the same time) and enables scaling the final system (massive applications with a huge number of users and/or agents).

**Fig 1:**
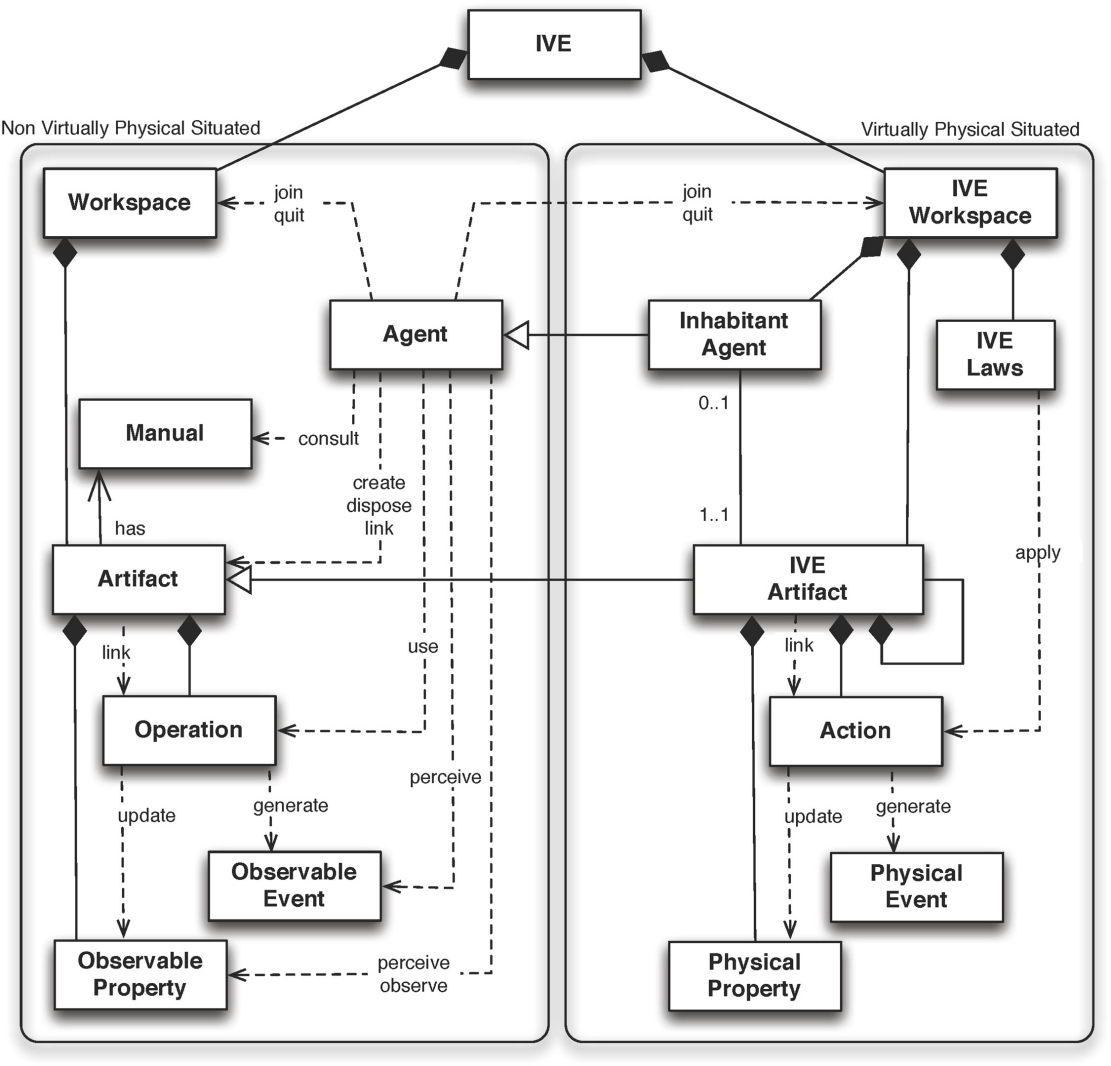
MAM5 Meta-model.

This model classifies the entities in the design into two sets (as seen in Fig 1). The first set is related to all the entities that do not have a physical representation in the IVE (non-virtually physically situated), while the second set is formed by all the entities having a representation inside the IVE (virtually physically situated). Inside the former set there are agents, artifacts, and workspace—in accordance with the A&A definition. In a similar way, inside the last set are IVE artifacts and inhabitant agents in the virtual environment (in fact, the inhabitant agent will have an IVE artifact representing its body in the IVE), and IVE workspace, representing the virtual place, and the laws defining and governing these places.

The MAM5 meta-model enables a differentiation between virtually represented and non-virtually represented entities, and also incorporates the definition of physical restrictions and properties in the modelling of the environment and inhabiting entities, respectively. The designer may define the different IVE laws governing the IVE workspaces (representing the physical laws of the real world) and may also define the different physical properties of the entities populating the virtual environments (including mass and length).

Some of these physical properties are very difficult to simulate or need numerous calculations. Moreover, it is desirable to design very complex simulations and link the simulation to a real environment to produce augmented worlds, or even mirror worlds. To this end, the *MAM*5 meta-model is modified by adding two new properties as will be explained in Section *MAM5 and JaCalIVE Extended*. To deal with this information, the concept of *smart resource artifact* is proposed to be incorporated into the meta-model.

### JaCalIVE (Jason Cartago implemented intelligent virtual environment)

Various approaches have recently been explored for using MAS as a paradigm for modelling and engineering IVEs. However, several difficulties remain: low generality and reusability; and weak support for handling fully open and dynamic environments where objects are dynamically created and destroyed.

**Fig 2:**
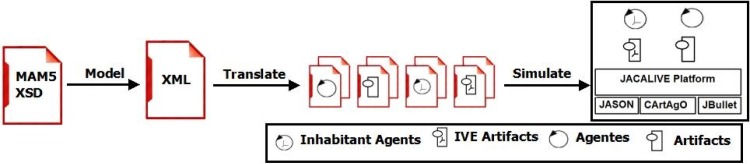
JaCalIVE General scheme.

The *JaCalIVE* framework is based on the MAM5 meta-model and was developed to tackle these difficulties. It provides a method to develop this type of application along with a supporting platform for execution. Fig 2 shows the steps for developing an IVE according to the *JaCalIVE* framework.

1. Model: the first step is to design the IVE. *JaCalIVE* provides an XSD based on the MAM5 meta-model. Using this approach, an IVE can be composed of two types of workspaces depending on whether they specify the location of the entities (IVE_Workspaces) or not (Workspaces). The specification of agents, artifacts, and the norms that regulate the physical laws of the IVE workspace is also included.
2. Translate: the second step is to automatically generate code templates from the design. This code is generated by parsing the XML file where the design is stored. One file template is generated for each agent and artifact defined in the model. As the XML file only includes a description of the agents and artifacts in the system, the template files generated include creation, accessing and communication between agents and artifacts, but they do not include any specific application code, that must be filled by the designer after the compilation process. Nevertheless, the creation of these files facilitates very much the IVE creation process. All the agents generated in this process are rational agents based on JASON, and the generated artifacts representing the virtual environment are based on CArtAgO. When the developer completes these templates with the application specific behaviour, the IVE is ready to be executed.
3. Simulate: Finally the IVE is simulated. As shown in Fig 2, *JaCalIVE* platform uses JASON, CArtAgO and JBullet. JASON offers support for BDI agents that can reason regarding their beliefs, desires, and intentions. CArtAgO offers support for the creation and management of artifacts, and JBullet offers support for physical simulation. *JaCalIVE* platform also includes internal agents (JASON based) to manage the virtual environment.

### Accessing the Environment

Among the distributed systems that interact with the real world, sensors and actuators are the physical interface [36] with the environment. Initially, sensors send data to clients in the same format that the data was acquired; for example, an RGB camera sends a frame with the acquired bitmap. Currently, the power the embedded systems (such as Arduino [37], Raspberry PI [38] or Beaglebone [39]) provides to sensors and actuators offers the possibility of transforming the raw data into specific information. When sensors or actuators provide processed data the device is usually called a smart device. Originally, in [40] Schmidt considers a smart device as *a device that is not ignorant about its environment*. When a smart device works in a distributed system, it is usually contextualized in a communications client/server paradigm. In [41], Salzmann defines a smart device as a device *where more intelligence is placed at the server side and little or no assumption is made about the client*. Some examples of smart devices are the Microsoft Kinect or the Asus xTion [42]. These kinds of devices provide images enriched with depth information directly from the server (smart device) to a client.

Currently, when a smart device offers more than the sensor information (or provides more than access to an actuator) in a distributed system, the smart device becomes a smart resource [43] and is contextualized in a publish/subscribe [44] paradigm. Examples include a sensor that provides specific environment elements such as tables or chairs in a home environment and clients that request the service: *let me know when the smart resource has found a chair*.

If a distributed smart resource provides smart information, the environment must be defined in the same smart terms. Consequently, the model used to observe the environment is at the core of a good MAS, and so there is a considerable literature defining what is a smart environment [45] or how to define the sensors that observe the smart environment [46].

### Smart resources

**Fig 3:**
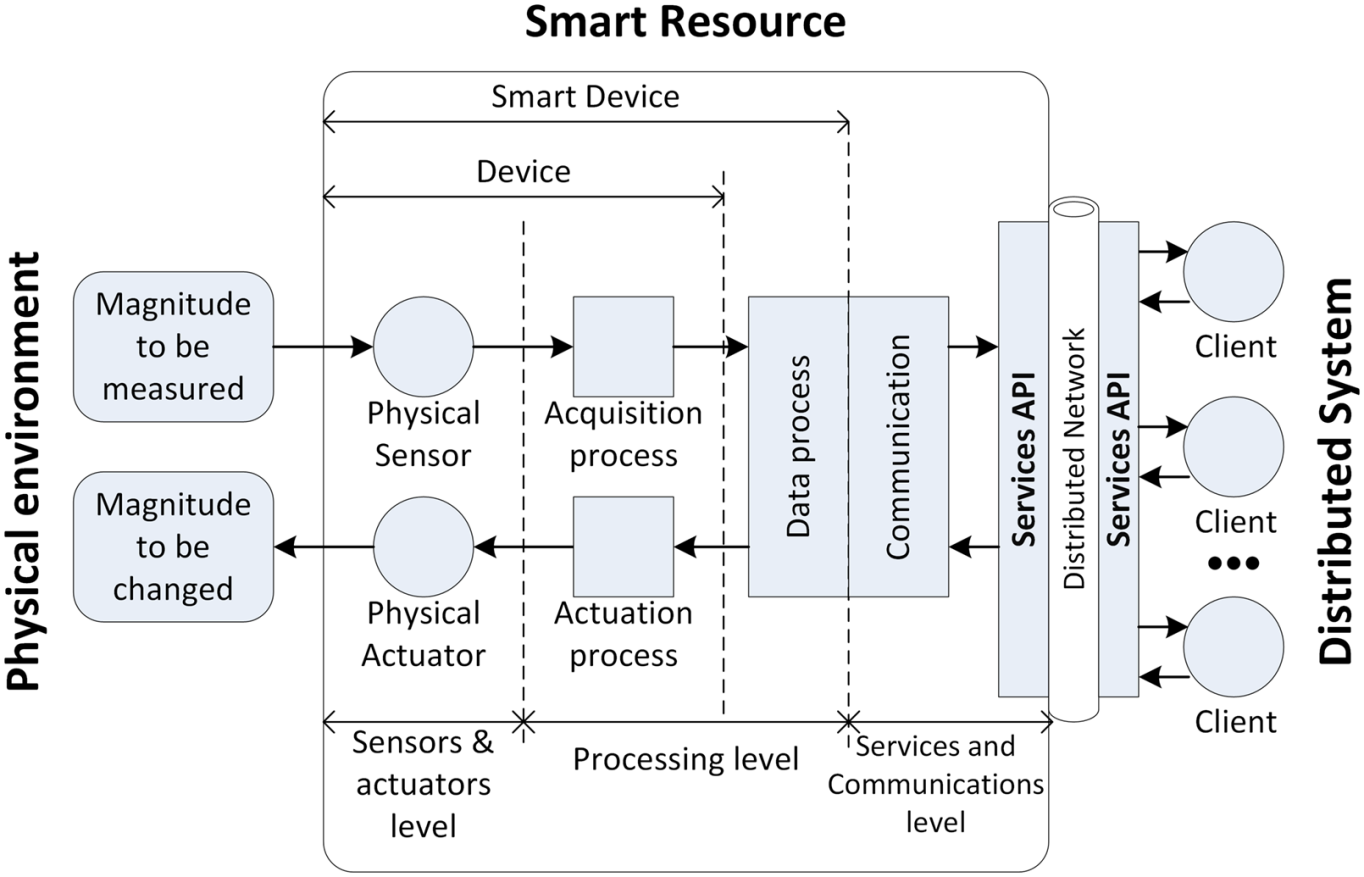
Components of a distributed smart resource.

Among the smart resources that operate in the real world, sensors read physical magnitudes and provide real information (from simple magnitudes such as temperature to complex information such as images acquired by an *RGB* camera). In the same way, actuators in the real world change physical magnitudes (or the resource characteristics): for example, by increasing the temperature or moving a resource. However, a smart resource provides access to this data and processes this data to produce richer information that is closer to the environment or client requirements. For example, a smart resource that offers the speed of detected objects in a bounded area, needs to detect the object and process data from various sensors by applying equations to calculate the speed. Smart resources must then publish the processing results in order for clients to receive the right information. Therefore, suitable and well known communication protocols must be used. Considering all the above, a distributed smart resource is structured in levels that convert it from a set of sensors or actuators to a resource with the capacity to process and distribute information. A distributed smart resource that is made up of three levels [47] is shown in Fig 3.

The sensor/actuator level provides basic functions for access to acquisition/actuation hardware. The processing level adds intelligence to the device so that it can locally transform data into useful information for the client. For example, when locating specific objects (people) within a specified distance in an RGBD frame [48], a smart resource is used for a specific function for a specific client. Beyond the specific smart resource function, when the information processed by the smart resource can change or clients can dynamically change their requirements, it is necessary to offer the information as services by means an application program interface (*API*) and a protocol. In these last cases, the use of a smart resource concept is justified to discriminate between objects defined by clients [47].

## Methods

In this section two different extensions incorporated into the *MAM5* meta-model and into the *JaCalIVE* framework are presented. The first extension enables the *IVE* to connect with the real world, using physical artifacts to obtain information from the real world. By using these devices it is possible to build complex simulations, complement real sensorization with virtual information, or even enable agents to control different elements in the real world.

The second extension introduces the human in the loop, and enables humans to interact in both worlds (real and virtual)—meaning in the augmented or mirror world.

The rest of the section presents how these extensions were added to the *MAM*5 meta-model and to the *JaCalIVE* framework.

### Modified MAM5

The first approach of *MAM5* [3] was intended for an *IVE* designer who wants to design an *IVE* based on a multi-agent system. Therefore, the authors only took into account the separation between entities that have a virtual representation in the virtual environment (virtually physically situated) and the entities that do not have a physical representation in the *IVE*, that is, that are not situated (non virtually physically situated).

As commented previously, *MAM*5 has been extended for use in designing *IVEs* as well as augmented worlds, or even mirror worlds. For this reason, it has to initially include all the sensors and actuators that enable access to the real environment and so mix virtual and real environment data. Then, it is incorporated the human in the loop within this mix of virtual and real environments.

**Fig 4:**
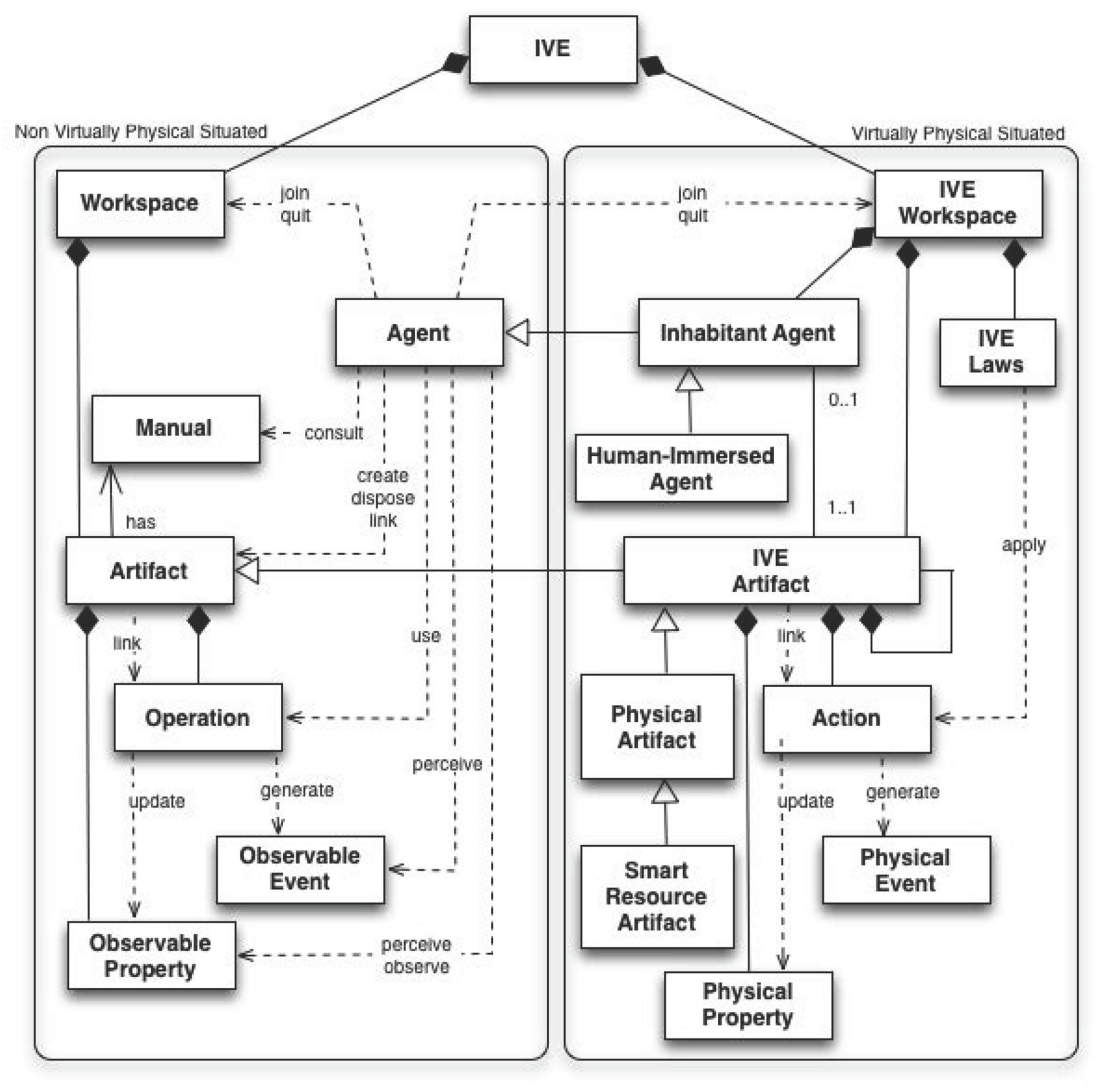
Modified MAM5.

These elements enable us to increase the possibility of creating an interaction between the real world with the *IVE* and the human with the agents—thereby creating a meta-model that enables the modeling of augmented worlds (Fig 4).

#### Human-immersed agents

This kind of agent models the representation of a human in the system. It can be simply an interface of the human with the MAS, or it can have the capability of perceiving and recognizing the associated human [49]. This agent may even model the preferences of the associated human (e.g. the music preference [50]).

To sum up, the main goal is to model the human in the loop, taking into account the double immersion—meaning humans interacting with agents with natural interfaces and agents interacting with humans as with other agents.

**Fig 5:**
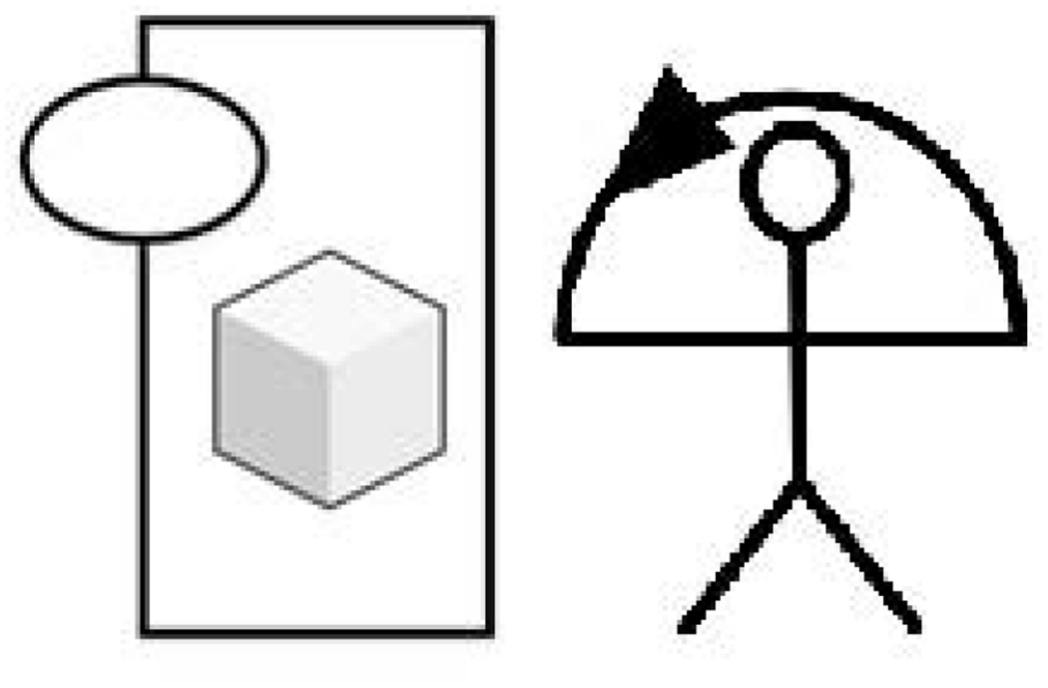
New symbols used in the *MAM*5 extension: smart resource artifact (left), and human immersed agent (right).

Fig 5 (right) shows the symbol used to model a human immersed agent.

#### Smart Resource Artifact

A smart resource artifact (SRA) is an artifact that incorporates embedded sensors and actuators providing access to the environment in an MAS.

These artifacts must have advanced processing and communication capabilities to supply high-level information by abstracting agents from data acquisition processing and recognition mechanisms.

In the proposed extended meta-model MAM5, an SRA is represented with a new symbol(see Fig 5 left).

**Fig 6:**
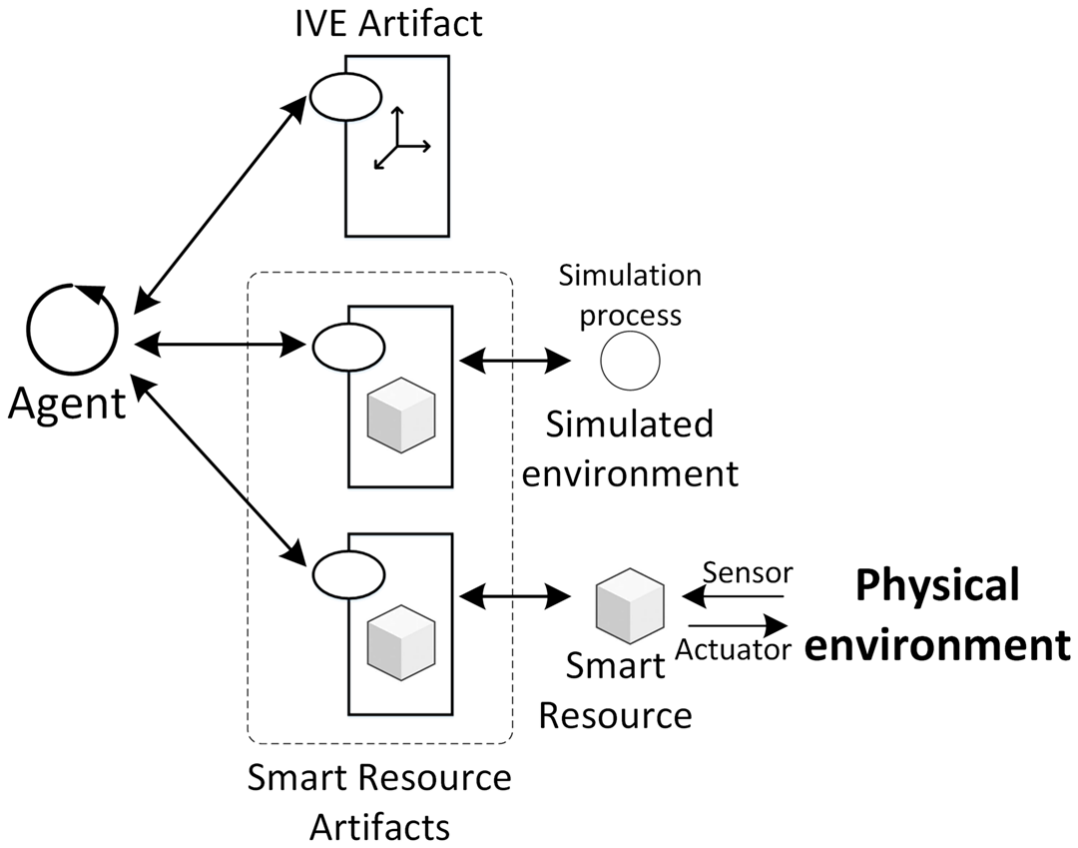
Interaction between agents and artifacts: IVE and SRA (simulated or a wrapper of a smart resource).

The functionality of an SRA can be provided by using a real device that works in the physical world. In this case, the SRA is a wrapper for the device. Additionally, this functionality can be simulated (Fig 6). Agents will not distinguish this characteristic and will access both SRA through the same interface. In this way, agents can use an SRA without knowing the source of the data (real or simulated).

When the SRA represents a real device, this device is implemented according to the smart resource described in the introduction (see sub-section *Smart Resources*). In this case, the SRA can be considered as a client of the smart resource in a similar way to clients appearing in Fig 3. The smart resource and its wrapper (smart resource artifact) communicate using a communications protocol based on services.

### JaCalIVE Modified

**Fig 7:**
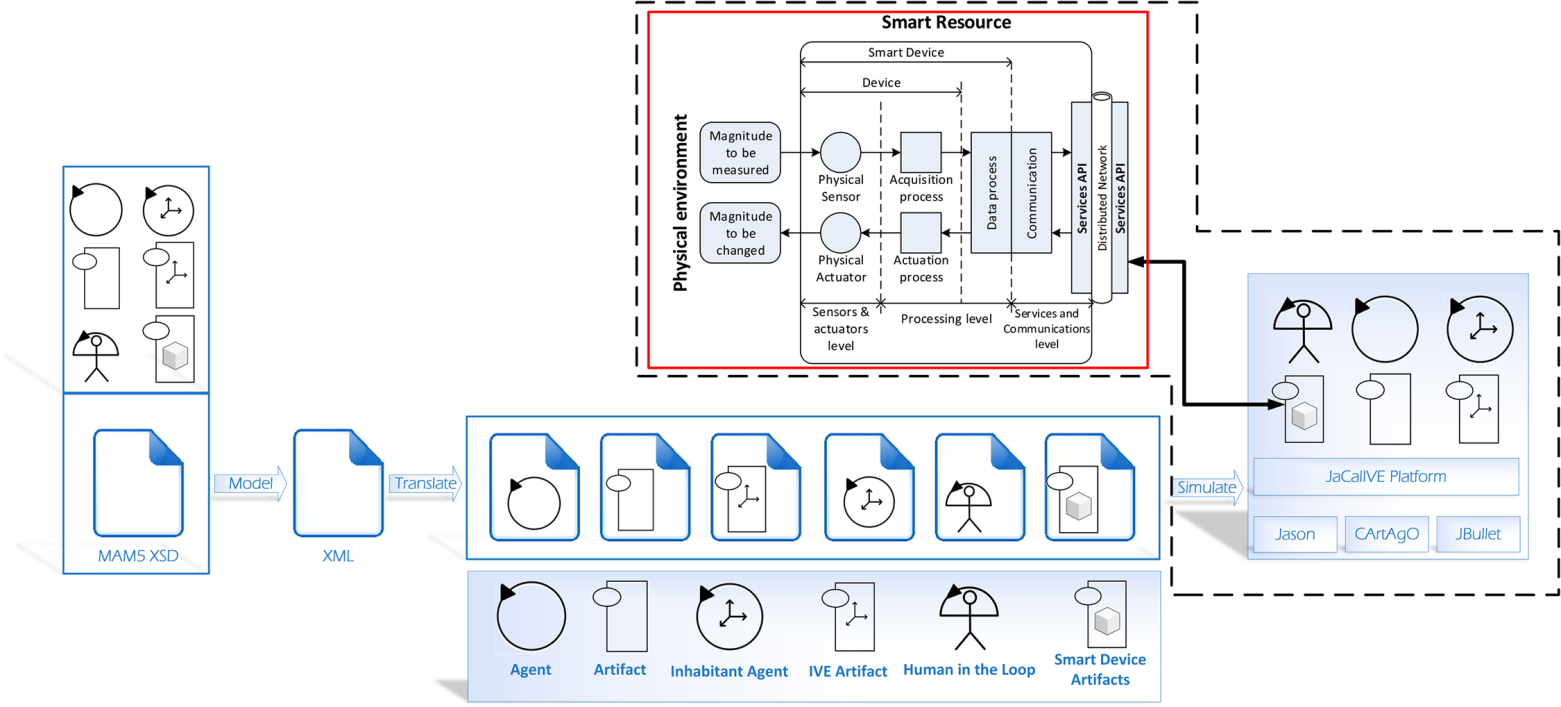
Modified *JaCalIVE*.

In this section the extension of *JaCalIVE* is presented to give support to the new modified *MAM*5 introduced in the previous section. These new elements enable the designer to connect the developed system to the real world and introduce humans in the same design. Using these new elements, it is possible to create a complex simulation, introducing measures such as temperature, pressure, biomedical signals, and other values. This measures will be used as perception of agents, and the agents can give an answer or change their behavior. Fig 7 shows the general scheme of modified *JaCalIVE*. Applications are developed in the same way as the previous version of *JaCalIVE*. The main difference is that there is a template to model the peculiarities of human immersed agents, and a new kind of artifact enabling access to the real environment (*SRA*).

**Fig 8:**
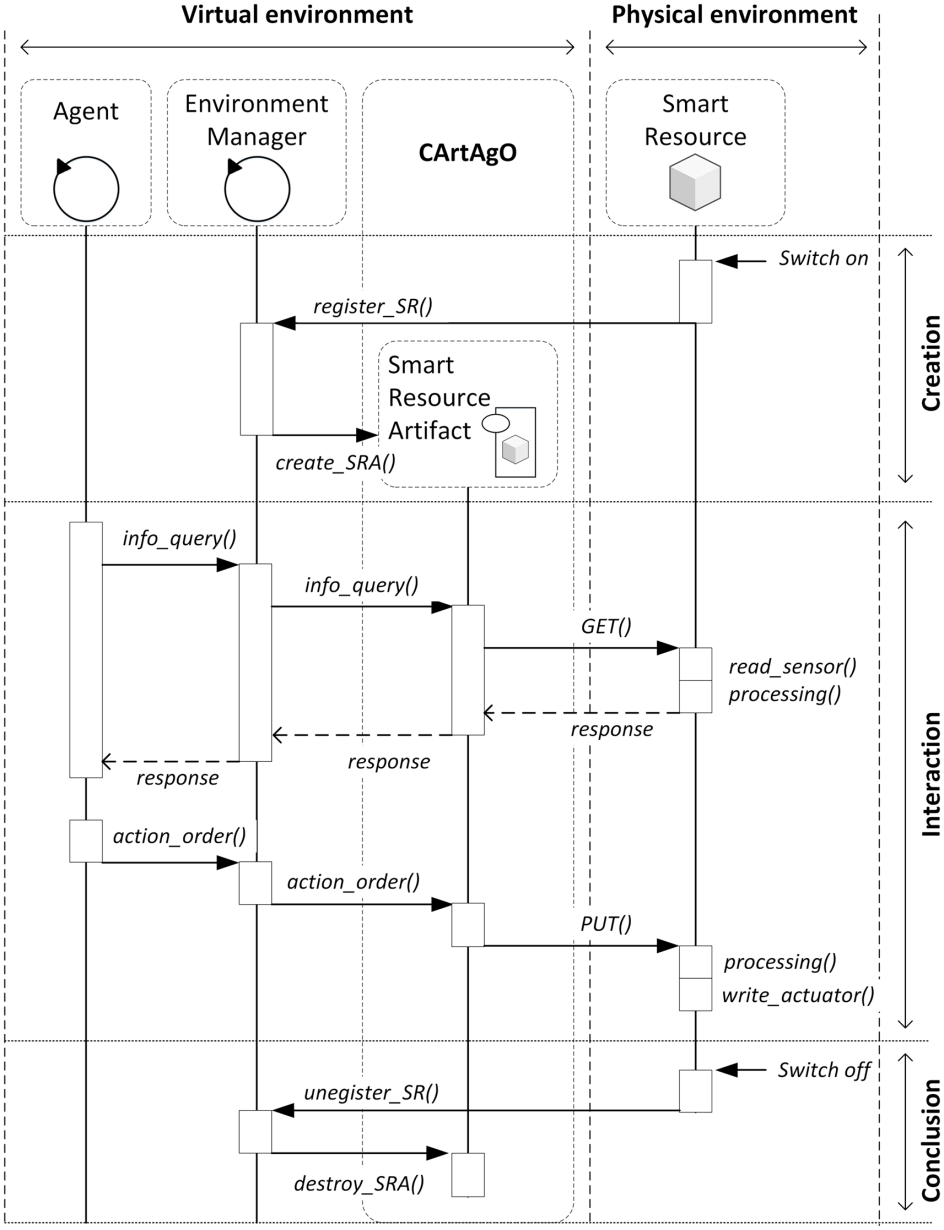
Protocol to work with a SRA based on a smart resource.

This access to the real world is possible when the SRA is connected to a smart resource (that is, the functionality of the SRA is not simulated). Communication between the SRA and the smart resource is based on access to services. The smart resource offers an interface to the services as used by its SRA wrapper in the system generated by the new *JaCalIVE* by following a proposed protocol (Fig 8).

When a smart resource is switched on, it initializes sensors, actuators and communications and then connects to the environment manager in order to register itself in the virtual environment. For that, SRAs need only know the IP address of the environment manager that is public in the system. Once connected, the smart resource sends to the environment manager an XML message with content about its identification, location, and the resources or services it offers. The environment manager then creates a smart resource artifact associated with the smart resource.

**Fig 9:**
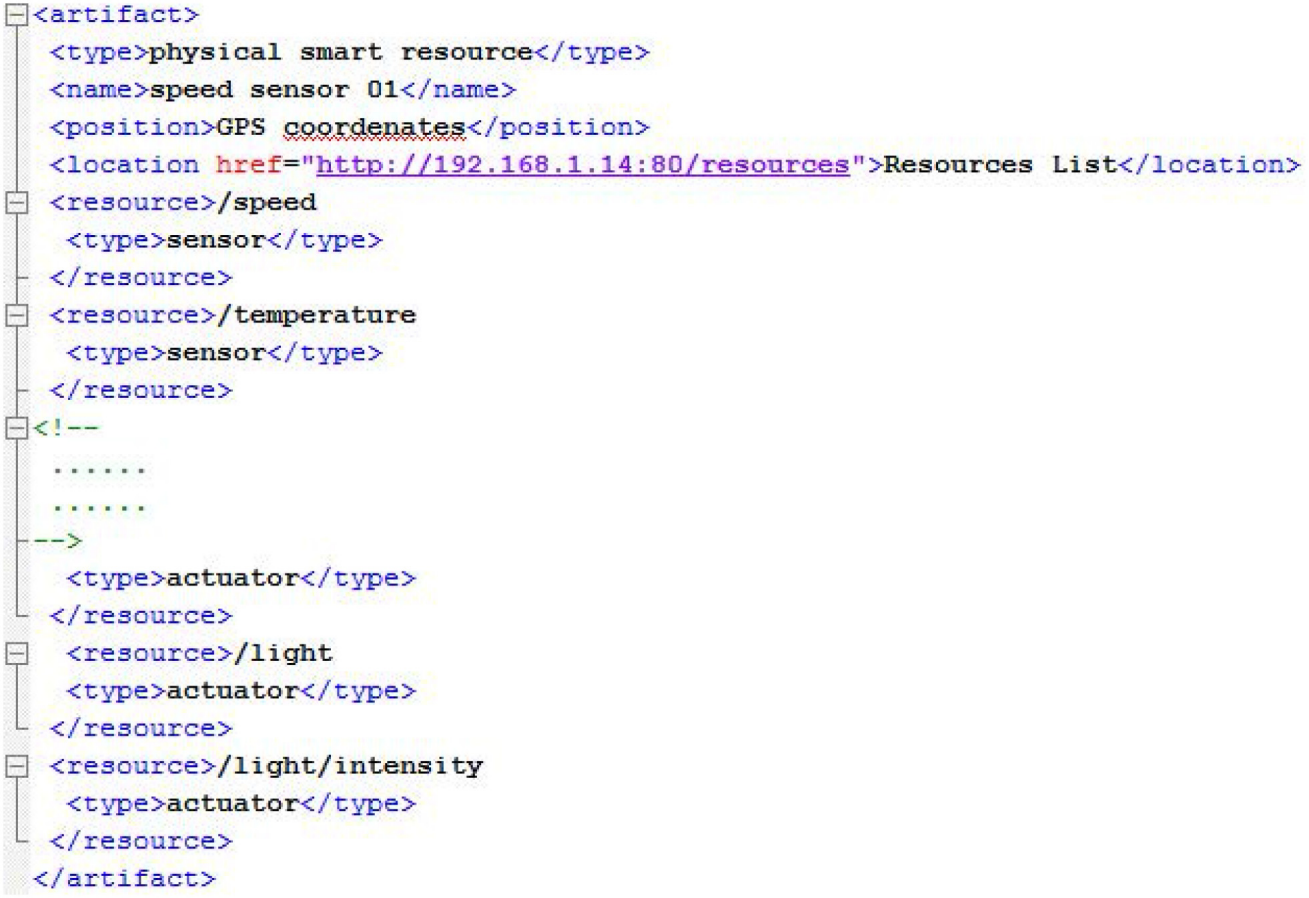
XML message to register a smart resource in the virtual world.

With the structure of the XML message (Fig 9), the environment manager knows how to access the resources/services offered by the registered smart resource. The *location* tag indicates the base URI (uniform resource identifier) of the smart resource and each *resource* tag identifies a different resource/service. A resource/service can be applied for by adding to the base URI its identifier. For example, the temperature could be obtained with: “http://192.168.1.14/resources/temperature”. Every resource/service has its own URI in the same way that representational state transfer (REST) services work (the software architectural style of the World Wide Web) [51].

SRAs work with resources/services through standard HTTP operations such as GET (to obtain the value of a resource: for example, the current temperature from a sensor) or PUT (to send data to update a resource: for example, to switch on/off a light actuator).

When an agent needs to access a resource, the agent makes a request to the environment manager that knows the associated SRA. The environment manager translates the request to the corresponding SRA and then this SRA uses the resource URI by sending a GET or PUT operation to the smart resource.

**Fig 10:**
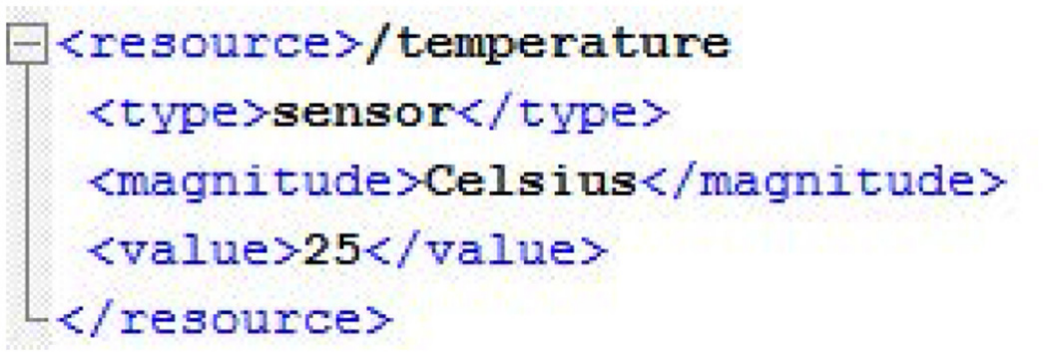
XML response message for GET operations.

**Fig 11:**
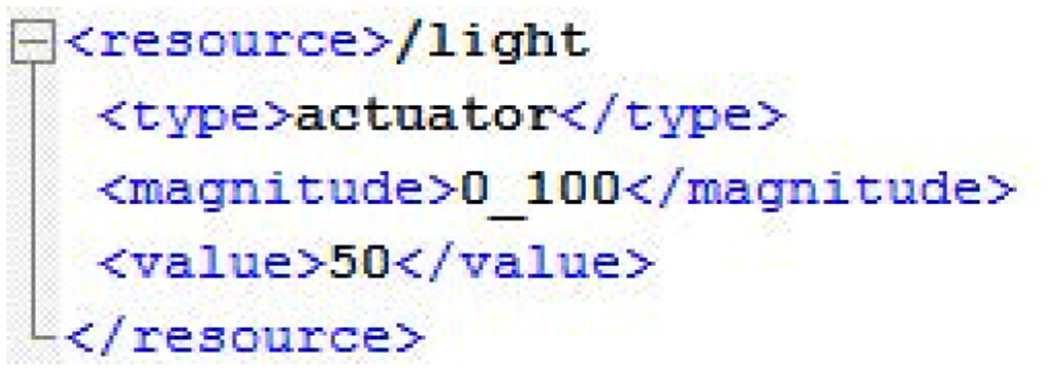
XML message for PUT operations.

In the case of a GET operation, the smart resource responds to the SRA with the content information of the resource by means of an XML message (Fig 10) and, in the case of a PUT operation, the SRA sends to the smart resource an XML message (Fig 11) with the information to be updated.

**Fig 12:**
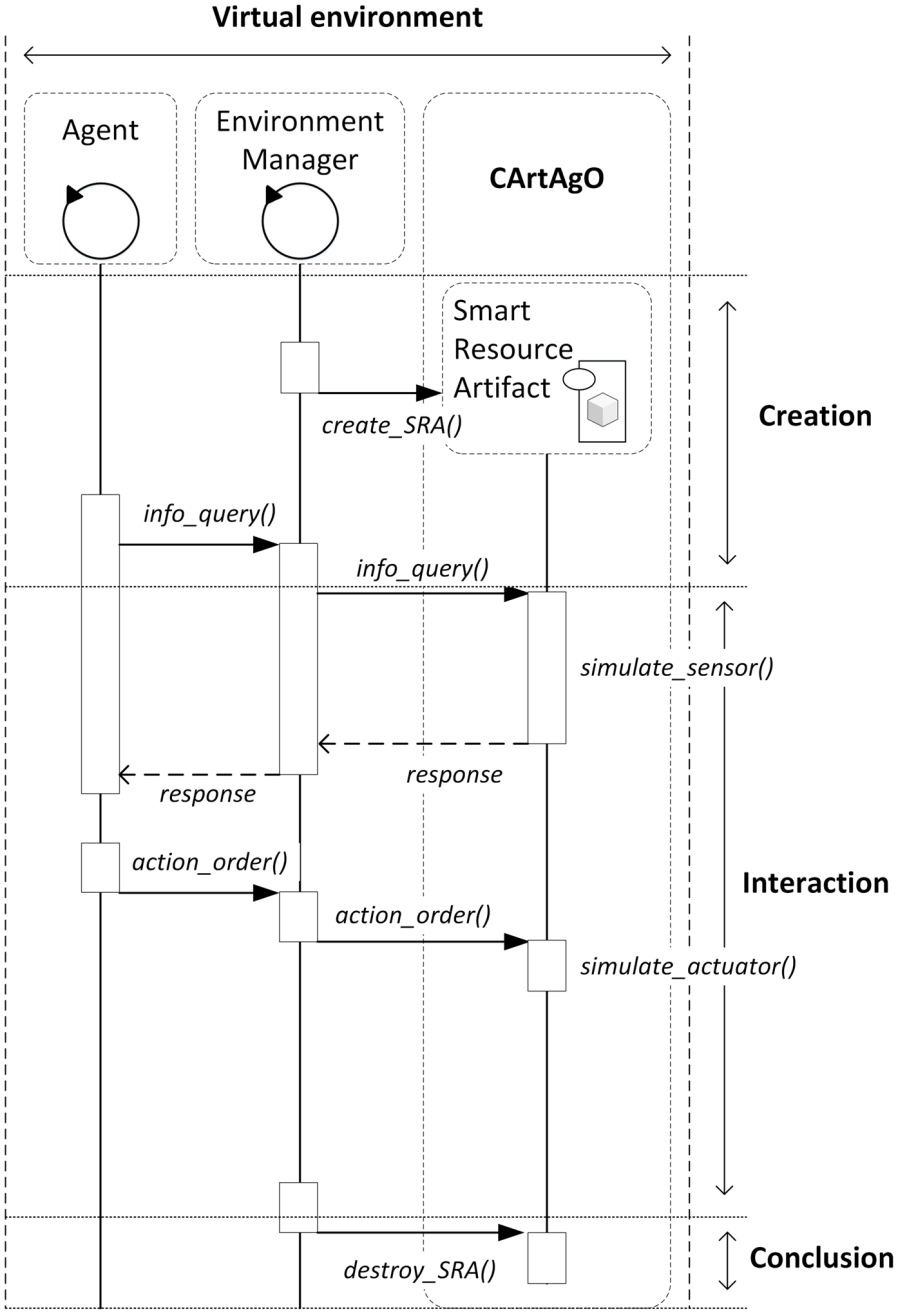
Protocol to work with a simulated SRA.

When the SRA is simulated (there is no smart resource associated) the protocol with agents (Fig 12) is similar to that presented previously. In this case, the environment manager creates the SRA depending on the system configuration. This SRA will simulate the smart resource functionality. To interact with the SRA, agents use the same protocol and interface as when a smart resource is associated with the SRA.

## Results

To validate the proposal, the extended JaCalIVE framework has been tested in a scenario for managing streetlights. Streets are populated with a very large number of streetlights. Even if these streetlights are using LED lights, the total energy consumption may be very large. One solution is the automation of the on and off switch using detection sensors. The position of such sensors is usually based on the experience of the person making the installation. This leads to number of sensors being higher than necessary. Moreover, the installation of such sensors is progressive and very slow. The proposal presented here uses the extended JaCalIVE framework to enable a reduction in the number of sensors needed and the installation time (mixing real with virtual sensors). Using agents to control the streetlights on/off can help improve user comfort, as they may control how many streetlights are switched on so that the user always has the sensation of being in a fully illuminated environment.

**Fig 13:**
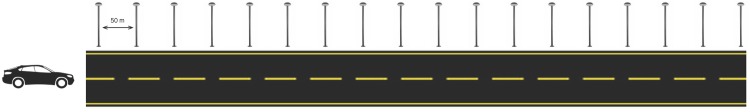
Prototype streetlights distribution.

More specifically, the proposed prototype is formed by a set of 18 streetlights distributed as can be seen in Fig 13. The lighting power of these streetlights can be automatically adjusted by the system. The main goal of the proposed scenario is to provide the appropriate light to any car driving along the street while maintaining the highest possible number of streetlights off or with the lowest possible power. The state of the streetlights must be adapted as a consequence of car displacement. As a car is moving along the street, the system must adapt the street lighting without disturbing the drivers.

**Fig 14:**
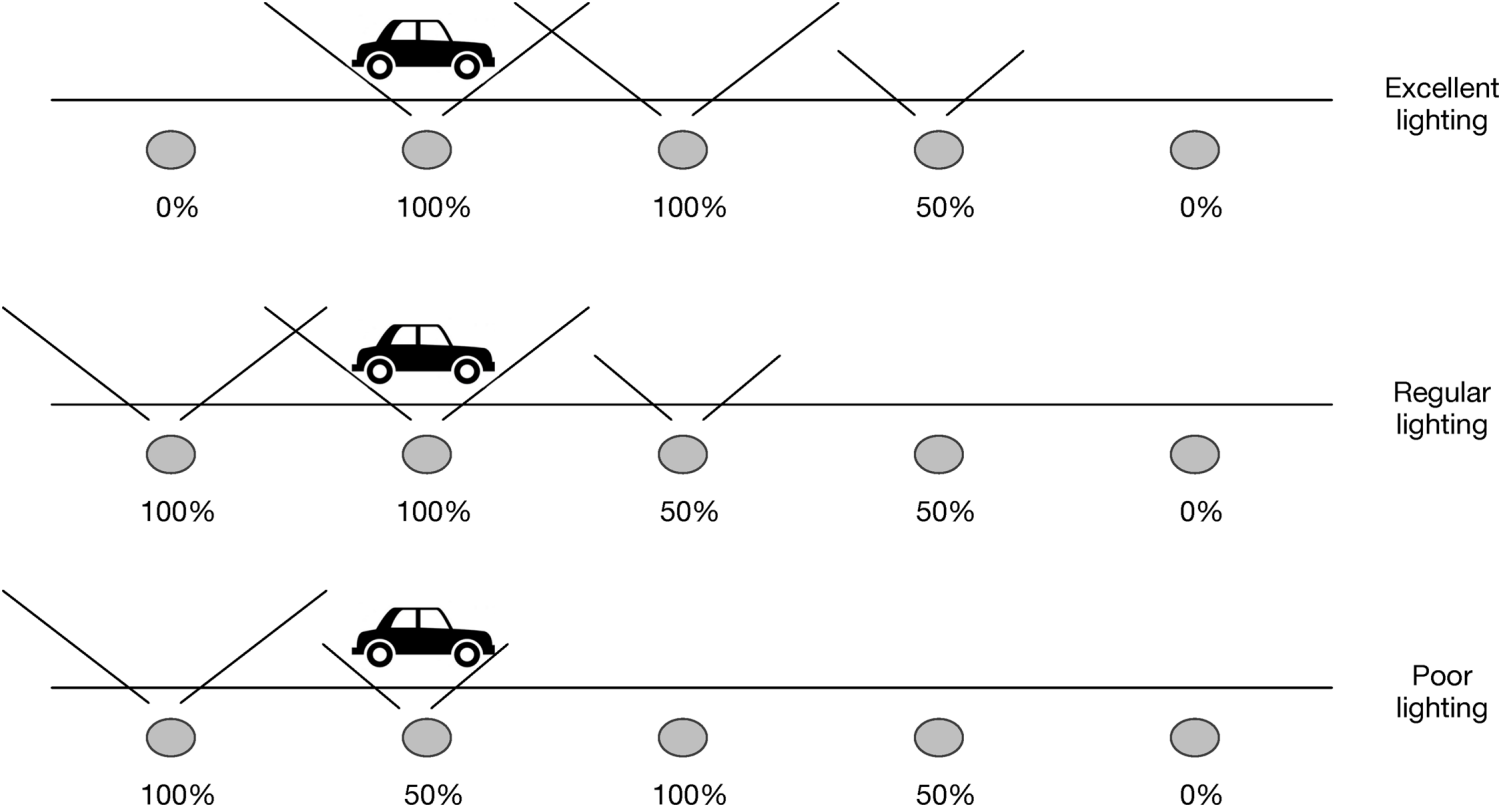
Prototype description view.

Variations of this scenario can be observed in Fig 14. The first situation is the preferred one because it provides excellent lighting to the front of the car. The second and third situations can be considered adequate from the point of view of light consumption, but the driver does not have an adequate view of the street. Any other combination with more streetlights on is discarded due to its higher power consumption.

### System design

**Fig 15:**
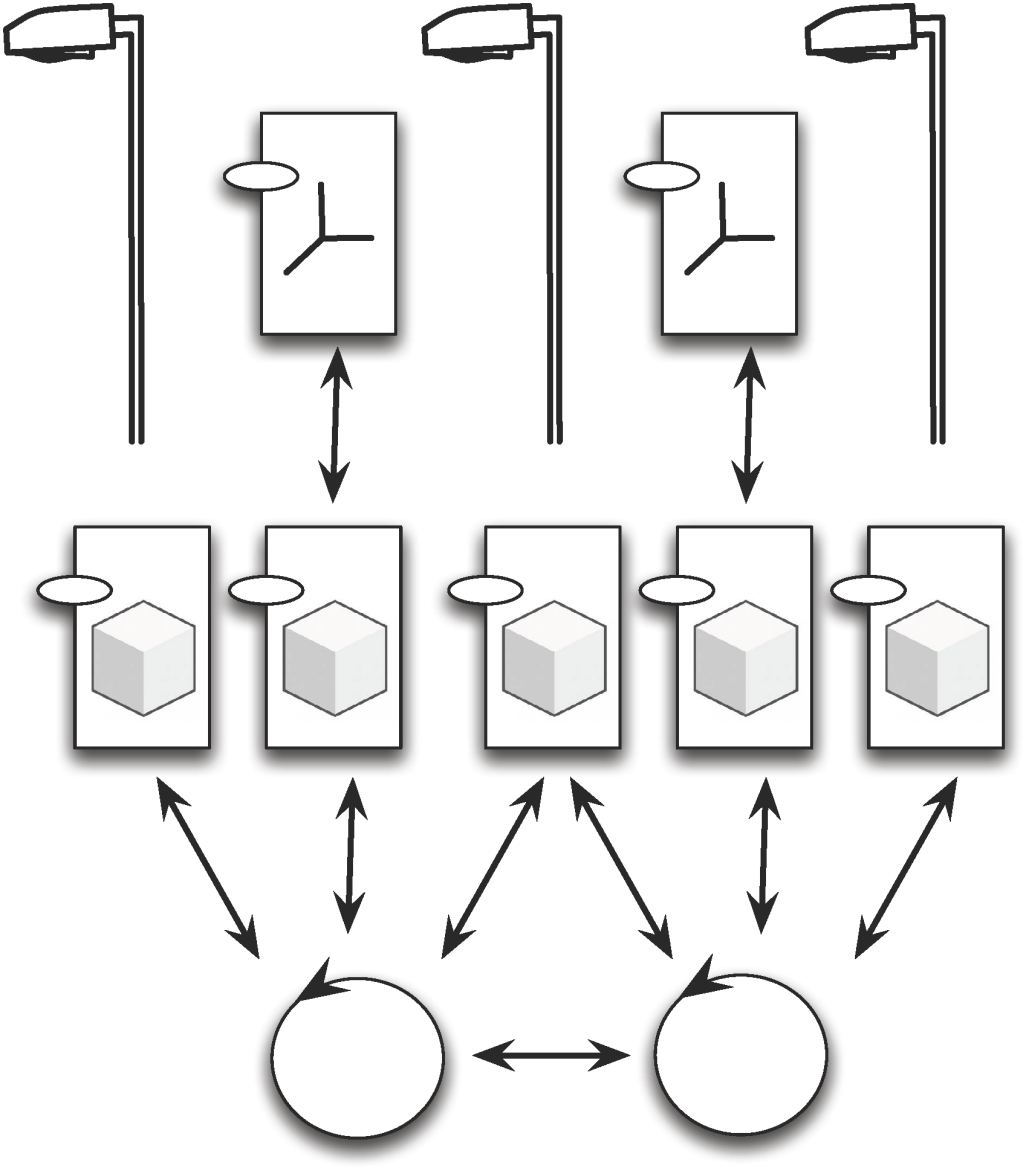
Model design for the streetlight system.

To develop the system described in the previous section, the following model using the extended *MAM5* meta-model has been devised (see Fig 15):

- Each car is modelled as an *inhabitant agent* taking into account that it can decide to follow or not a constant velocity, increase or decrease its velocity, or even stop at any time.
- Each streetlight will be modelled as a *Smart Resource Artifact*, although there are two types: some being only a wrapper for a real smart resource (with an actuator to switch on/off the streetlights and presence sensors to detect real cars and their velocity), and those in which the sensorization part is simulated (by means of an *IVE artifact*).
- A set of agents, each in charge of controlling n streetlights. This number, along with the number of real sensors, constitutes the two parameters that could be used to tune the system. In the prototype it has been used n = 3.

It is important to underline that the topology of the street map will influence in the minimum number of real sensors needed to have a good enough system performance. For instance, if there is a streetlight close to a crosswalk, this streetlight would need a real smart resource, because it is necessary to detect if the car stops (due to people crossing).

Each *smart resource artifact* detecting a car would inform the agent (or agents) it is linked to that a car is passing and its speed. As the goal of the system is to obtain a lighting similar to the first situation in Fig 14, agents must communicate that a car is approaching and at which speed. Following the example of the design in Fig 15, if a car is detected by the sensor on the left streetlight in the figure (assuming that in streetlights the figure represents real sensors, and *IVE artifacts* represent simulated sensors) it communicates this information to its agent (on the left) along with the car speed. The agent sends the order to this same streetlight and to the next to light at full power, and to the third streetlight to light at half power. The car speed is used to simulate when it is going to arrive at the next streetlight (that does not have sensors). When this occurs, the agent orders the streetlight to light at full power (and to the previous streetlight to turn off) and the agent also sends to the third streetlight the order to change to full power and communicates with the right agent to send it the car position and speed so that this agent can turn on the corresponding streetlight to half power. This process will go on for each car passing along the street. The *Smart Resource Artifact* is in charge of each streetlight and responds to the order that provides the most light if various orders are received simultaneously.

#### Smart resource artifacts

To control lighting in smart cities [52], current systems usually work with basic sensors that detect people or vehicles and send their values directly to an automatic control system that switches on/off the corresponding lights. To improve the data obtained from sensors, it is proposed to work with smart resources that provide processed information. A smart resource connected to various sensors sends the values of the sensors (such as the presence or not of an object) and processes these values to provide richer information. A smart resource could measure the speed of the object detected, its acceleration, size, color, and could even identify the object.

Furthermore, in the case of the detection of vehicles, a smart resource could also report on the number of vehicles detected by measuring the density of traffic. Therefore, light control agents will have more information to adapt the number of streetlights switched on, their duration, and intensity to the traffic circumstances.

**Fig 16:**
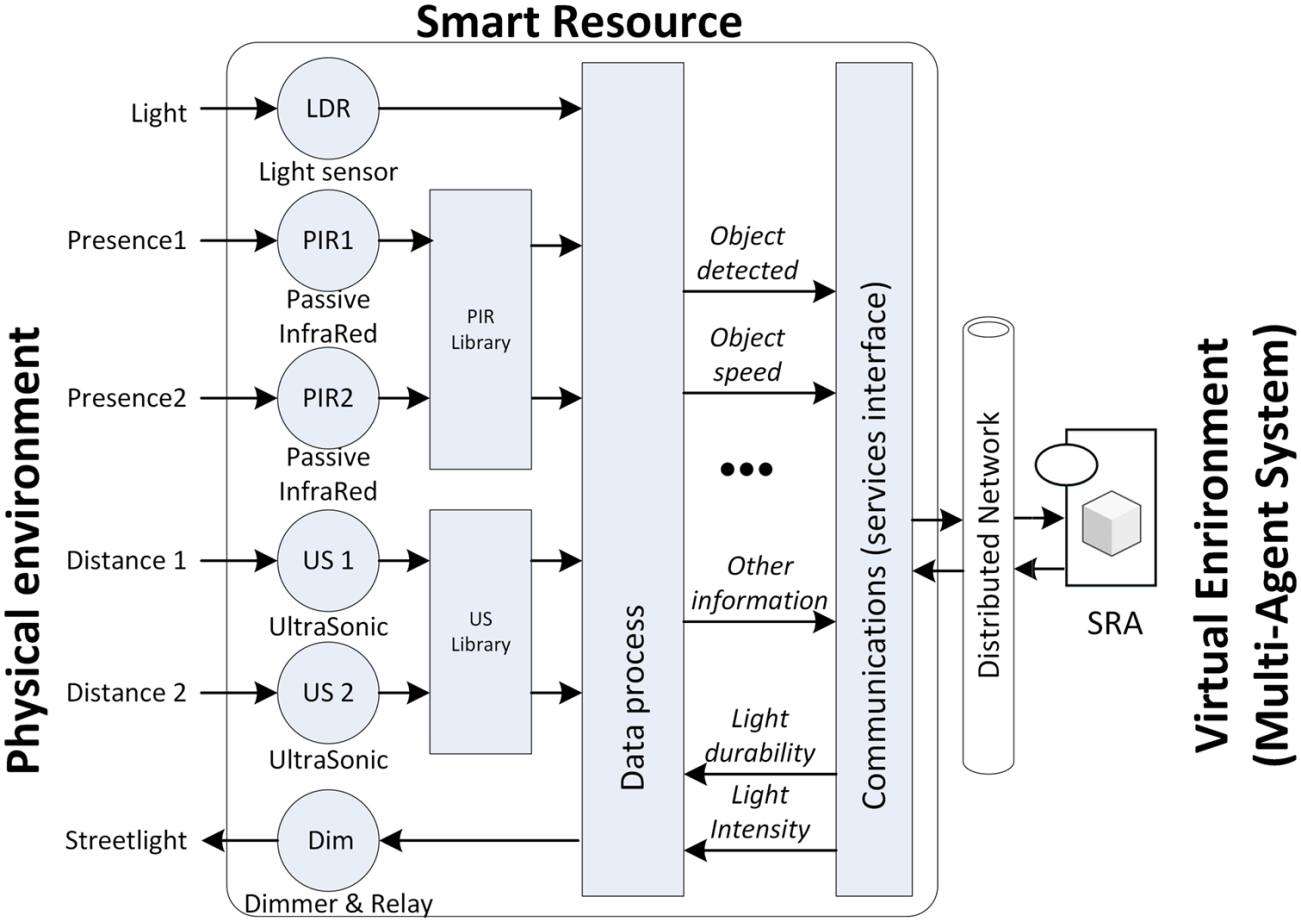
Internal components of the speed detector and light control smart resource.

In the experiments presented in this paper it is necessary to provide the speed of the detected objects and control the light intensity of the streetlights. Fig 16 shows the design of the smart resource used.

To detect objects, the smart resource uses a passive infra-red (PIR) sensor. When the PIR detects an object, two ultra-sonic (US) sensors, placed in a row, start to measure the speed. The processing level detects the time when the distance obtained in every US sensor is reduced by the presence of the object. The object speed is then calculated by taking into account the temporal difference. Depending on the resources/services required by the agents, other information, such as object length, may be sent.

Agents control the streetlights connected with the smart resource. Consequently, the smart resource also offers the resources/services: light intensity and durability.

### Implementation

#### Agents and artifacts developed

In the developed prototype (see Fig 13), a street with 18 streetlights was controlled. Each of these streetlights is controlled by a smart resource artifact. There are nine agents in the system, each in charge of three smart resource artifacts, taking into account that two agents may share a smart resource artifact as indicated in Fig 15.

These agents and artifacts have been defined according to the Modified *MAM*5 implementation in the *JaCalIVE* modified framework. The system is defined in an XML file that is compiled to the Jason agents and CARTAGO artifact templates that is filled with the specific code for the system.

**Fig 17:**
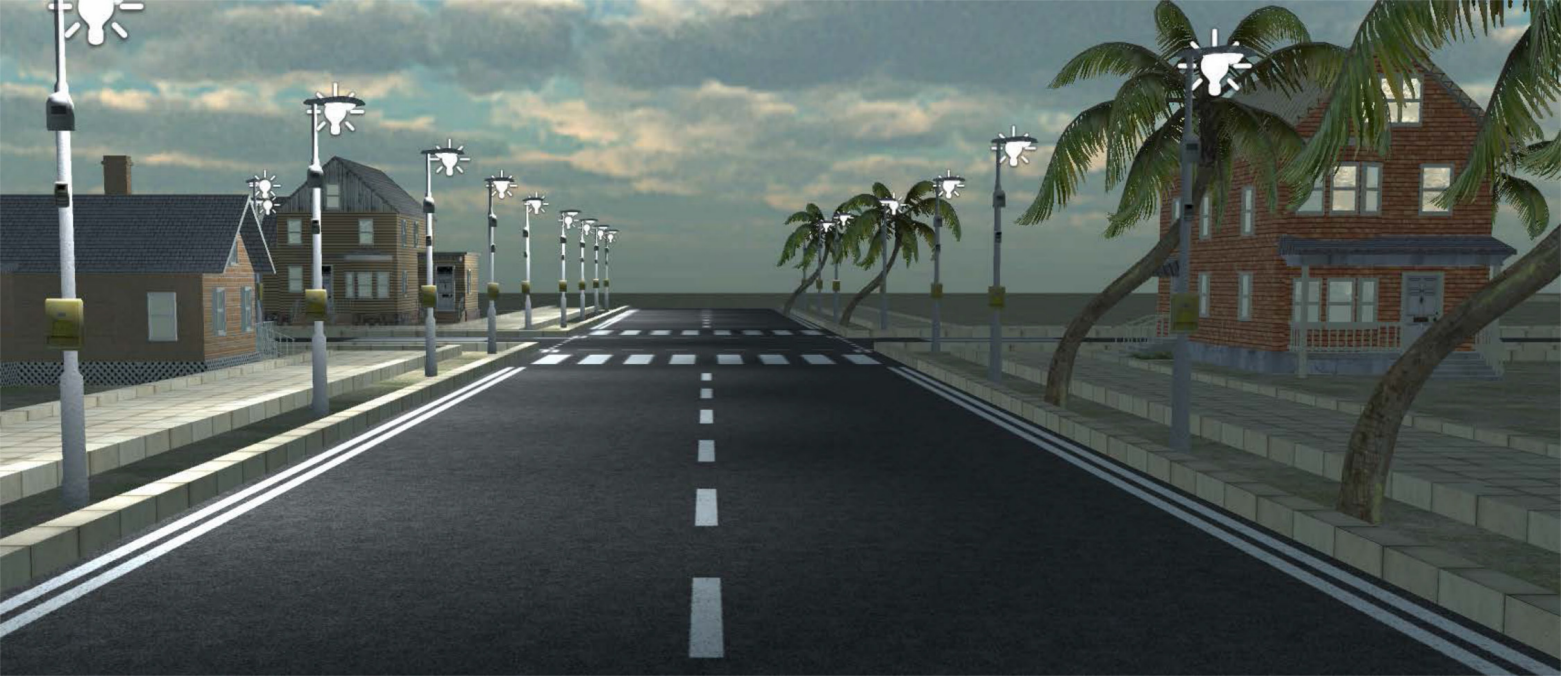
Developed prototype.

To interact with the simulation, a Unity3D render engine have been developed as can be seen in Fig 17.

#### Smart resources developed

**Fig 18:**
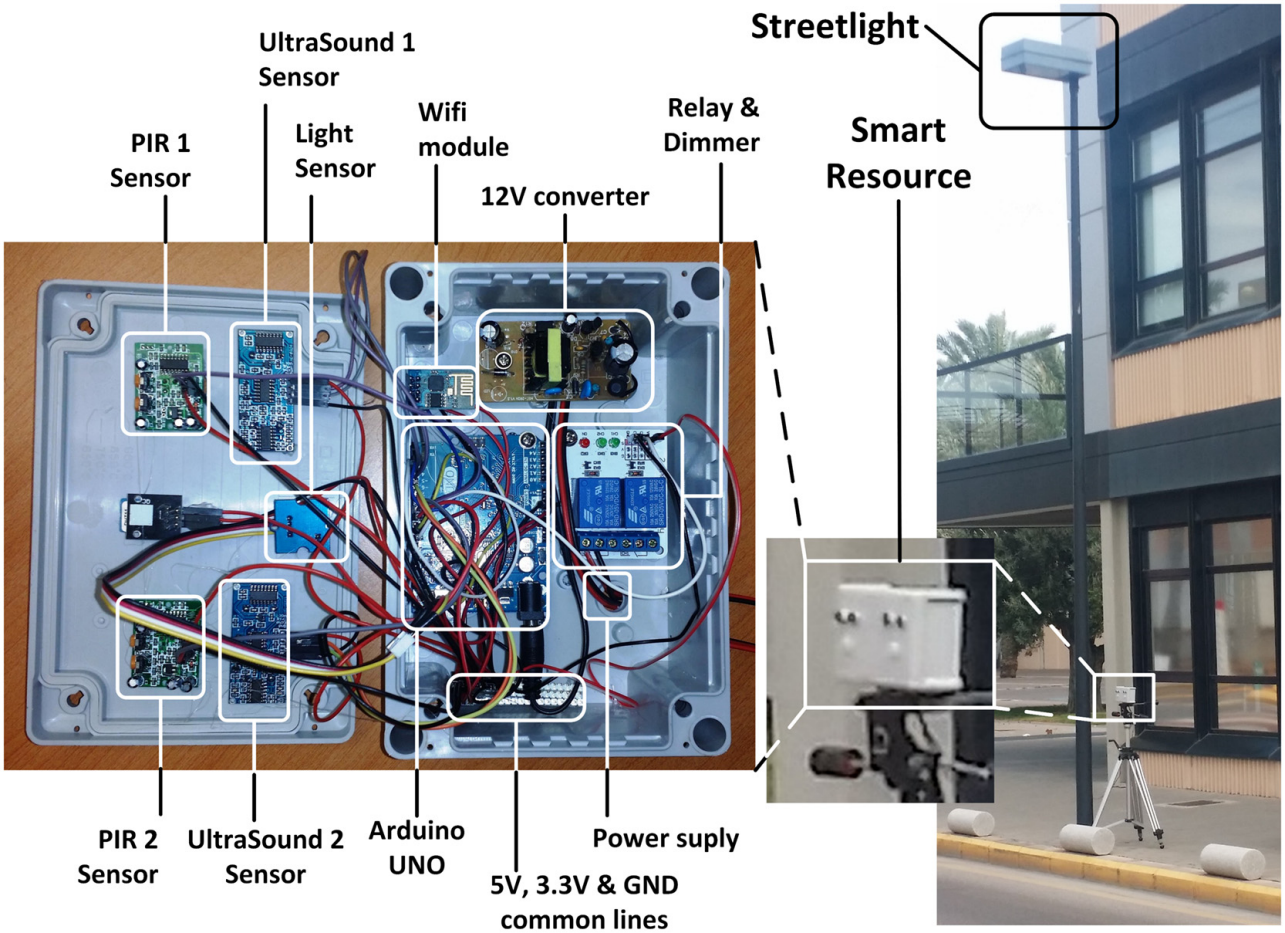
Prototype developed of the smart resource.

Smart resources are mounted on a waterproof case in order to validate them in external environments. All sensors, actuators, and control components, are placed inside the case (Fig 18). The following components have been used:

- Sensors
  - PIR sensor GH-718, it is an infra-red sensor typically used in smart illumination systems [53].
  - US sensors HC-SR04, used in a wide variety of systems due to its accuracy [54] and speed [55].
  - The light sensors used are LDR (Light Dependent Resistor) with dual operational amplifier (LM358) to increase the accuracy of the signal and adapt it to be used in external environments [56].
- Actuators
  - Relay SRD-05VDC, a classical relay used in control systems [57].
  - Dimmer: a PWM-based LM3405, that was used previously in light control systems [58].
- Control and communications
  - Arduino UNO, one of the most used micro-controller board, based on the ATmega328P and very suitable to implement devices in Agents systems [59])
  - Wifi module, the recently appeared ESP8266 that uses IEEE 802.11 standard protocol, and it is becoming popular including in critical systems [60].

SRAs connect with smart resources through a client/server communications system based on TCP/IP network. This communications system uses wireless connections accomplishing the standard IEEE 802.11b in the 2.4 GHz band and 11 Mbit/s. *JaCalIVE* Framework includes a TCP server that allows smart resources to be registered according to the previously defined protocol. Besides, every smart resource configures a TCP server that is executed in the Arduino micro-controller in order to attend SRAs requests based on the XML messages described in the protocol.

### Execution tests

Different tests have been done in order to evaluate the proposed framework. Specifically, the aim of the tests is to validate the use of smart resource artifacts as a way to model smart resources in the *JaCalIVE* framework. Moreover, the experiments allow studying the minimum set of real sensors that the proposed system needs to ensure a specific error margin.

In the proposed model, a real sensor in every streetlight won’t be necessary if agents can simulate the movements of the cars correctly. For that, the multiagent system in the virtual environment will have to get the positions of the cars by using both virtual and real smart resource artifacts (that is, connected to a simulation or to a real smart resource). Logically, real measurements will come from the real smart resource artifacts, that are connected to available smart resources, and the virtual smart resource artifacts will have to estimate their measurements.

The next graphics show the effect produced in the simulation when the number of real versus virtual smart resource artifacts is increased. The results are obtained when the car moves with different speeds and speed-ups. Every graphic shows the error in the simulation by calculating the difference between the estimated position of the virtual car and the position of the real car when this goes through every streetlight.

To do this, the framework has been tested changing the number and the position of the real sensors.

- Experiment 1: There only exist two real smart resources, they are connected to streetlights number 1 and number 18. The rest of lampposts are controlled by virtual sensors.
- Experiment 2: Streetlights number 1, 9 and 18 are connected to real smart resources. Similar to the previous experiment, the rest of lampposts are controlled by virtual sensors.
- Experiment 3: Streetlights number 1, 6, 12 and 18 are connected to real smart resources. As before, the rest of lampposts are controlled by virtual sensors.

Moreover, each experiment has been evaluated with different behaviors of the drivers:

- Behavior A: the car speed is constant all along the experiment.
- Behavior B: the car initially accelerates and then it maintains its velocity constant.
- Behavior C: the car initially accelerates and then it decelerates its velocity.
- Behavior D: the velocity changes in a random pattern.

**Fig 19:**
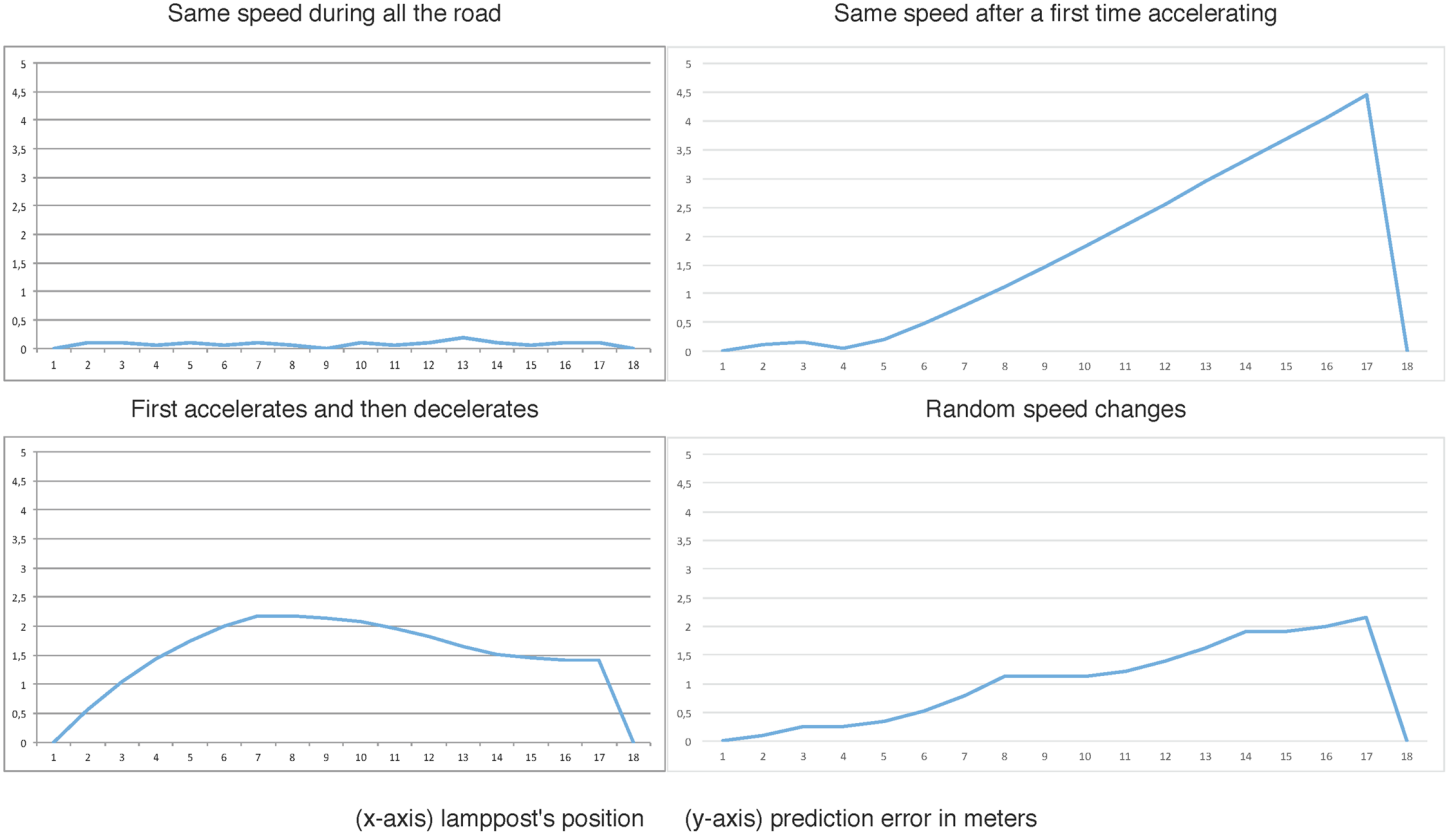
First experiment: Smart Resources only in 1st and 18th lampposts.

Fig 19 shows the results of the Experiment 1 with the four different commented behaviors. As we can see, except from the situation where the driver maintains the same speed, results show a high error prediction done by the virtual sensors. This is logical because they only have information about the speed of the car supplied by the real sensor placed on lamppost 1.

**Fig 20:**
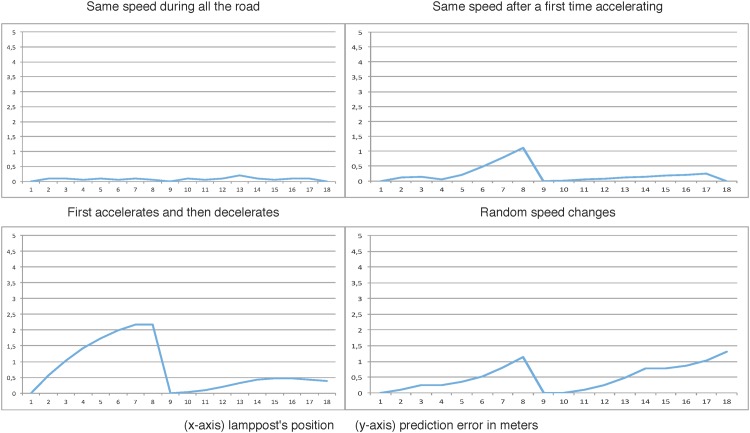
Second experiment: Smart Resources in 1st, 9th and 18th lampposts.

Fig 20 shows the results of the Experiment 2 with the four different commented behaviors. In this situation, the error prediction has been reduced in all the situations. Nevertheless, the error is still high in some situations where the car changes the speed for a longer time.

**Fig 21:**
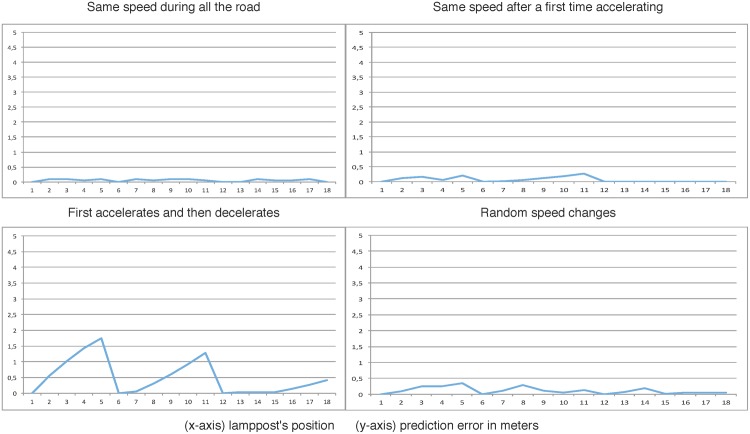
Third experiment: Smart Resources in 1st, 6th, 12th and 18th lampposts.

Fig 21 shows the results of the Experiment 3 with the four different commented behaviors. In this last experiment, we can see how the predictions done by virtual sensors have been improved in all the situations independently of the driver’s behavior.

Logically, when the number of real sensors is increased, the error in the simulations is reduced. As it can be seen in Fig 21, with only 4 out of 18 real smart resources the error obtained even in behaviours that are difficult to predict as the third one, the error obtained is admissible to be used as a solution for incremental installations. So, in this case, the automatic streetlight system could be working with an admissible error in 1/4 of the whole time needed for the installation of the whole real smart sensors. It’s important to remark that minimizing the error in the prediction of virtual sensors, the system achieves a correct lighting of the road employing the minimum set of real sensors. This is a promising result that leads to go on working in this line of developing.

## Conclusions

A new approach for the design and implementation of mirror worlds has been presented in this paper. This new approach is an extension of the *MAM*5 meta-model and the *JaCalIVE* framework. The goal of these new extensions is to include the human in the loop and connect (even in real-time conditions) the physical environment with the virtual world—and so enable the creation of augmented worlds. Specifically, the aim was the aggregation of new components such as *smart resources artifacts* and *human immersed agents* in the development process of these intelligent virtual worlds, and help designers create *irror worlds*.

The integration of these elements represents a great advantage because it facilites the design of models that can capture the real world with a high level of abstraction, and so enable the designer to create a representation of the real world that facilitates the introduction into these models of components based on AI and MAS technology. Apart from providing a new method to develop this type of intelligent virtual world, the proposal enables the execution of the system through the use of a supporting platform.

Finally, an application example of the proposed framework was presented. This example is related with the efficient and intelligent use of streetlights. The example has been used in a satisfactory way to obtain a suitable prototype using the proposed models and execution framework.

The method presented can be used to determinate the minimum set of real sensors that a system needs to ensure a determinate error margin.

As a future work, the aim is to continue developing more complex examples, integrating other sensors or actuators with new utilities that produce more information about the environment and enable the control of complex physical devices. This framework can be applied to many different domains. Some of them, that we would like to underline, include big systems simulations in ambient intelligence to predict the energy saving reduction in smart cities and smart mobility. Moreover, applications in the smart health domain where agents could be used to monitor bio-signals, falling detection, and so on. This monitorization could be done not only by software agents but also with mobile robots. In the robotics domain, this framework can be used to model and simulate the real environment using smart resources to improve the accuracy of the robot navigation.
